# Distance Dependent Contribution of Ants to Pollination but Not Defense in a Dioecious, Ambophilous Gymnosperm

**DOI:** 10.3389/fpls.2021.722405

**Published:** 2021-09-08

**Authors:** Adriana Aranda-Rickert, Javier Torréns, Natalia I. Yela, María Magdalena Brizuela, Verónica S. Di Stilio

**Affiliations:** ^1^Centro Regional de Investigaciones Científicas y Transferencia Tecnológica de La Rioja (CRILAR-CONICET), Anillaco, Argentina; ^2^Universidad Nacional de La Rioja, La Rioja, Argentina; ^3^Department of Biology, University of Washington, Seattle, WA, United States

**Keywords:** protective mutualism, ant pollination, dioecy, *Ephedra*, gymnosperm pollination, pollination drop, wind pollination, Gnetales

## Abstract

Dioecious plants are obligate outcrossers with separate male and female individuals, which can result in decreased seed set with increasing distance between the sexes. Wind pollination is a common correlate of dioecy, yet combined wind and insect pollination (ambophily) could be advantageous in compensating for decreased pollen flow to isolated females. Dioecious, ambophilous gymnosperms *Ephedra* (Gnetales) secrete pollination drops (PDs) in female cones that capture airborne pollen and attract ants that feed on them. Plant sugary secretions commonly reward ants in exchange for indirect plant defense against herbivores, and more rarely for pollination. We conducted field experiments to investigate whether ants are pollinators and/or plant defenders of South American *Ephedra triandra*, and whether their contribution to seed set and seed cone protection varies with distance between female and male plants. We quantified pollen flow in the wind and assessed the effectiveness of ants as pollinators by investigating their relative contribution to seed set, and their visitation rate in female plants at increasing distance from the nearest male. Ants accounted for most insect visits to female cones of *E. triandra*, where they consumed PDs, and pollen load was larger on bigger ants without reduction in pollen viability. While wind pollination was the main contributor to seed set overall, the relative contribution of ants was distance dependent. Ant contribution to seed set was not significant at shorter distances, yet at the farthest distance from the nearest male (23 m), where 20 times less pollen reached females, ants enhanced seed set by 30% compared to plants depending solely on wind pollination. We found no evidence that ants contribute to plant defense by preventing seed cone damage. Our results suggest that, despite their short-range movements, ants can offset pollen limitation in isolated females of wind-pollinated plants with separate sexes. We propose that ants enhance plant reproductive success via targeted delivery of airborne pollen, through frequent contact with ovule tips while consuming PDs. Our study constitutes the first experimental quantification of distance-dependent contribution of ants to pollination and provides a working hypothesis for ambophily in other dioecious plants lacking pollinator reward in male plants.

## Introduction

Dioecious plants, where female and male reproductive structures are produced on different individuals, are prone to pollen limitation (Wilson and Harder, 2003; Schlessman et al., 2014). Since these obligate outcrossers are incapable of selfing, pollen must be transferred from male to female plants by wind or via biotic vectors. The distance between individuals within a population is a major factor affecting pollination and seed production in dioecious plants (de Jong et al., 2005). Pollen flow decreases with increasing distance from the pollen source in wind-pollinated species (Robledo-Arnuncio and Gil, 2005; Bittencourt and Sebbenn, 2007), and distance can also influence insect-pollinated species due to pollinator behavior favoring nearby sources (de Jong et al., 2005). Most pollen flow occurs over short distances because of either gravity in wind-pollinated species (Di-Giovanni and Kevan, 1991), or nearest-neighbor pollination in animal-pollinated species (Proctor et al., 1996; Tambarussi et al., 2015).

Most gymnosperms are dioecious (64% of extant species, Walas et al., 2018), and many depend on the wind for pollination (Faegri and van der Pijl, 1979). Female cones (megasporangiate strobili) produce pollination drops (PDs), ovular secretions that capture airborne pollen and draw it into the ovule, where fertilization takes place (Gelbart and von Aderkas, 2002). Both insect pollination and ambophily (pollination by both wind and insects) have been documented in the order Gnetales (Pearson, 1909; Kato et al., 1995; Bolinder et al., 2016), where PDs also function as a reward for pollinators (Nepi et al., 2017). In ambophilous, dioecious plants insects may enhance pollination success compared to an exclusively anemophilous pollination mode, especially in environments where conditions favoring either wind or biotic pollination vary spatially and temporally (Stelleman, 1984).

*Ephedra* (*Ephedraceae*, Gnetales) are dioecious shrubs or vines of semi-arid ecosystems (Ickert-Bond and Renner, 2016). Pollination by animals (mostly insects, but also lizards) has been described for at least four Mediterranean *Ephedra* (Porsch, 1910; Bino et al., 1984a,b; Meeuse et al., 1990; Rydin and Bolinder, 2015; Bolinder et al., 2016; Celedón-Neghme et al., 2016; Fuster and Traveset, 2019). *E. foeminea* is so far the only exclusively animal-pollinated species producing PDs in male cones, from abortive ovules (Bolinder et al., 2016), in addition to in female cones. Other *Ephedra* are ambophilous, with PDs produced by both sexes (Bolinder et al., 2016; Celedón-Neghme et al., 2016), or only in female cones (Meeuse et al., 1990). Having morphologically bisexual male cones with sterile, PD-producing ovules that attract pollinators is considered an ancestral trait, while most derived species (including those in the South American clade) have lost this character (Bolinder et al., 2016). Hence, an open question is to what extent insects that feed only on female PDs contribute to pollination, and whether they enhance seed set in ambophilous *Ephedra* lacking reward in males. Alternatively, PD secretion dynamics could play a role, given that certain gymnosperms, including at least one species of *Ephedra* (Moussel, 1980), replace PDs after removal by insects until the ovule is fertilized (Owens et al., 1980; Tomlinson et al., 1997; Mugnaini et al., 2007). Ongoing secretion of PDs following removal by putative insect pollinators might scavenge pollen they left behind on the micropylar tube (the external extension of the entrance to the ovule) (von Aderkas et al., 2018), while further enhancing insect visitation via an increased offer of reward. Hence, pollinator behavior is likely an important determinant of pollination success in systems where repeated foraging on PDs enhances the probability of pollen grains being drawn into ovules and achieving fertilization.

Ant-plant mutualistic interactions are frequently mediated by sugary reward (Rico-Gray and Oliveira, 2007). The most widespread form of ant-plant mutualistic interactions are defensive mutualisms, where ants consume extrafloral nectar (EFN) and protect plants from herbivores (Marazzi et al., 2013). Ant pollination, where floral nectar is offered as a reward in exchange for the pollination service, is rarer (e.g., Peakall and Beattie, 1991; Del-Claro et al., 2019; Delnevo et al., 2020). On the one hand, ants have been traditionally considered poor pollinators because of the presence of metapleural gland secretions on their integument that negatively affect pollen viability (Beattie et al., 1984) and due to their limited movement, since wingless foragers only visit resources near their nest (Faegri and van der Pijl, 1979; Domingos-Melo et al., 2017). On the other hand, not all ants have metapleural glands or negatively impact pollen germination (de Vega et al., 2009; Yek and Mueller, 2011), and certain angiosperms have been described as ant-pollinated (e.g., Gómez and Zamora, 1992; Carvalheiro et al., 2010; Ibarra-Isassi and Sendoya, 2016). Ants consume PDs and carry pollen in at least three Mediterranean species of insect-pollinated or ambophilous *Ephedra* (*E. aphylla, E. foeminea* and *E. distachya*, Moussel, 1980; Meeuse et al., 1990; Bolinder et al., 2016). However, these studies did not conduct ant exclusion experiments, hence the role of ants remains obscure. Moreover, even though the limited range and site-fidelity of ants (i.e., continuously returning to the same plant for a food, Hölldobler and Wilson, 1990) are traits that do not favor pollen transport between individuals of separate sexes in obligate outcrossers, this pollinator behavior could be adaptive in ambophilous *Ephedra* that produce PDs in females only.

Here, we investigate the interaction between ants and dioecious *Ephedra triandra* to address the following questions: (1) Is *E. triandra* pollinated mostly by wind, do ants contribute to pollination and, if so, is this contribution distance-dependent? (2) Do ants enhance plant fitness by defending seed dispersal units (cones) against herbivore damage (and is there a double mutualism)? To answer these questions, we characterized the insect visitor assemblage, quantified ant pollen load and pollen germinability, and set up exclusion experiments to assess the relative contribution of wind, winged insects and ants to seed production (as a proxy of pollination) and to seed cone damage. We also analyzed the effect of distance to the nearest male plant on seed set, estimated the amount of pollen grains reaching female plants by wind as a function of distance, and studied the dynamics of PDs secretion under laboratory conditions in relation to ant visitation rate. Our predictions include: (1) Frequent and persistent visitation of ants to female cones, but not to male cones, (2) Pollen transport by ants without a decrease in pollen viability, (3) Enhanced seed production with ant visitation, (4) Decreased pollen flow to females by wind with increasing distance to the nearest male, and (5) Increased contribution of ants to pollination and seed set with increasing distance to the nearest male (as a result of higher ant visitation to female cones). If ants were to contribute to plant defense, we expect that ant-excluded plants should exhibit higher seed cone damage or lower seed set and higher cone damage (in the case of a double mutualism), compared to open pollination treatments.

## Materials and Methods

### Study Area

Fieldwork was conducted in two seasons, between October and December 2019 and again in 2020 in Anillaco (28° 48′ S, 66° 56′ W; 1,400 m a.s.l.), La Rioja Province, northwestern Argentina. The vegetation type corresponds to the northern portion of the Monte Desert, an open shrubland dominated by *Larrea cuneifolia* (“jarilla,” Zygophyllaceae) and shrubby Fabaceae and Cactaceae (Abraham et al., 2009). The climate is semi-arid with marked seasonality, the average annual temperature is 16.9°C, and the average annual precipitation is 233 mm, falling during the December-March summer wet season (Anillaco Meteorological Station).

### Study System

**FIGURE 1:**
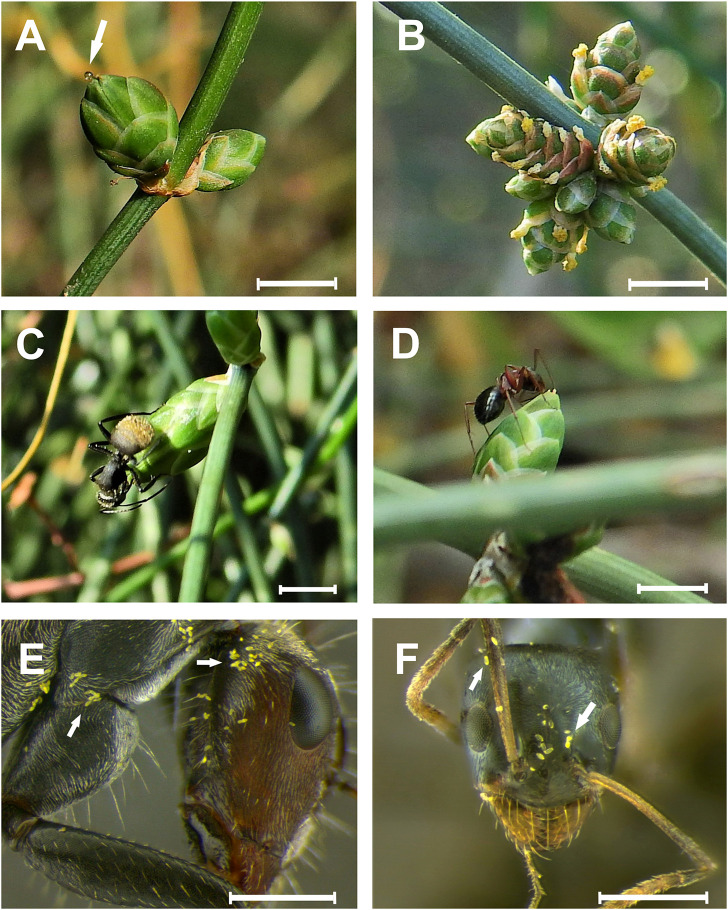
Reproductive structures and ant visitors of the dioecious gymnosperm *Ephedra triandra* (Gnetales). **(A)** Female cones, one with pollination drop (arrow); **(B)** cluster of male cones at anthesis; **(C)** *Camponotus mus*; and **(D)** *C. punctulatus* foraging on the micropylar tubes of the two ovules where pollination drops collect. Ants collected on female cones showing *E. triandra* pollen grains (white arrows): **(E)** *C. blandus*, with pollen on its head, mesosoma and legs, and **(F)** *Forelius chalybaeus*, with pollen on its head and antennae. Scale bars: **(A,B)** 5 mm; **(C,D)** 2 mm; **(E,F)** 0.5 mm.

*Ephedra triandra* Tul. (Ephedraceae) are perennial, dioecious shrubs up to 2 m tall that grow leaning on the vegetation, with highly reduced leaves and flexuous photosynthetic branches (Zavala-Gallo, 2016). This species is distributed in semiarid regions of south America, from Bolivia and southern Brazil to Central-Western Argentina (Zavala-Gallo, 2016). The female reproductive units are the megasporangiate strobili, or “seed cones,” consisting of bracts that start dry and green and become fleshy and red at maturity, with the distal pair enclosing two ovules that turn into seeds after fertilization. Each ovule integument extends into a micropylar tube that secretes a PD (Figure 1A; Kubitzki, 1990). The male reproductive units are the microsporangiate strobil, or “pollen cones” consisting of dry and green bracts, sterile at the base, followed by fertile bracts enclosing a stalked microsporangiophore each with pollen sacs (Figure 1B). *Ephedra triandra* pollen has the distinctive ellipsoidal, ridged and large (40.6 μm mean equatorial diameter) appearance typical of Gnetales (Bolinder et al., 2015), setting it apart from pollen of other taxa, especially since this is the only *Ephedra* growing in the study site. *E. triandra* is in pollination phase during Spring (October to early December); seeds mature and cones become red and fleshy from January to March.

### Pollination Drop Production

To determine whether pollen cones (from male plants) had abortive ovules that could lead to the production of pollination drops, their reproductive structures were collected from the field in November 2019, then observed and photographed under a dissecting microscope in the laboratory. To document PD production in the field, branches of five male and five female plants were isolated from ants and other insect visitors with mesh bags and a sticky resin (Hormigel^®^, Ecoworld, Argentina) applied onto a band of paper tape at the base of the branch. After 24 h, the production of PDs was observed with a 20x hand lens.

### Insect Visitation

The composition and abundance of the visitor assemblage was determined for 15 female plants by direct observation of seed cones at the green, pollination stage, between October and November 2019. Preliminary observations to assess the timeframe of insect visits were carried out in 2 h intervals throughout the day, and between 20:00 and 24:00 h at night. Based on these results, and since no nocturnal visitors were observed, we subsequently concentrated our observations during the day, from 0700 to 10:00 h and from 17:00 to 19:00 h. Insect activity is highest during these times in the Monte desert, where insects have adapted to avoid the warmest hours of the day (Aranda-Rickert and Fracchia, 2011a). The sampling procedure was to count all cone visitors in 5-min periods on each plant, for a total of 100 min of observation per plant (1,500 h in total). Any insects feeding on the PDs or contacting the micropylar opening of the ovules were considered cone visitors and hence potential pollinators. The relative abundance of cone visitors was the number of individuals of each species compared to the total number of visitors, and the frequency was the number of plants in which each visitor was recorded, over the total number of experimental plants. During each census, insect behavior was observed and photographed on site. Insects were then captured using nets and aspirators, placed individually in vials, and transferred to the lab for identification using taxonomic keys (Kusnezov, 1978; Fernández, 2003; Fernández and Sharkey, 2006). Insect visitors to female cones were examined under a stereo microscope Leica MZ12 to look for *E. triandra* pollen attached to their bodies and photographed with Leica Application Suite v 3.5.0. Voucher specimens of insect visitors were deposited at CRILAR Entomology collection (CRILAR-En-Ar).

### Exclusion Experiments to Determine Pollination Mode

To determine the relative contribution of winged insects, ants and wind to pollen transfer and seed set, we applied three treatments to randomly chosen branches on 15 female plants. To account for the influence of the distance between male and female plants, we selected female plants at variable distances from the nearest male, ranging from 0.1 m (intermingled plants) to 23 m. Female cones were in early stages of development by the end of September 2020 (green, before pollination). Treatments were: (1) “Open pollination,” in which branches are simply tagged and left open to the wind, to ants and to winged insect pollination; (2) “Ant exclusion,” in which cones are open to wind pollination and winged insects, but ants are excluded by the application of sticky resin. All branches and other vegetation in contact with the treatment branch that could act as aerial bridges for ants were removed; and (3) “Wind only,” in which cones are excluded from insect visitation (ants and winged), but wind-borne pollen is allowed. To this end, branches were covered with 0.4 mm mesh mosquito netting to exclude all winged insects while allowing wind-borne pollen, and sticky resin was applied to exclude ants. As control for seed set by apomixis (without fertilization) a total exclusion treatment was added by enclosing the branches in tightly woven cotton fabric bags and sticky resin to exclude airborne pollen, insects and ants. Prior to the onset of the experiment, we counted the number of cones on each experimental branch (60–85 cones/branch, total number of cones for each treatment on the 15 plants: Open pollination = 1,056, Ant exclusion = 1,086, Wind only = 1,052). Experiments were checked periodically (every 2 or 3 days) to ensure that ants were effectively excluded.

To control for the potential interference of mesh bags on pollen transfer by wind, we used pollen traps. Pollen traps consisted of pairs of glass slides coated with Vaseline on one side, one of them enclosed in the same mesh fabric used for the “Wind only” treatment, the other exposed. Four pairs of pollen traps were placed at a height of ∼1m on each of the 15 female plants, at each cardinal point. Pollen traps were set in November 2020, collected after 3 days, and screened under a microscope to count the number of *Ephedra* pollen grains per slide. During the selected days, the weather was dry, sunny and with low wind velocity, and male individuals were at anthesis.

Experimental and control branches were harvested after 6–8 weeks, at the latest possible stage of seed maturation (seeds of darker color and bigger size and bracts turning red) but before cone abscission, to avoid seed cone loss by gravity or animal dispersers. Seeds were dissected and examined under a stereo microscope to confirm the presence of healthy white embryos, as a proxy for seed viability. The number of mature seeds per treatment was used as an estimate of fertilization success and computed as the percentage of ovules maturing into seeds (relative to the initial number of ovules). The initial number of ovules was calculated by multiplying the initial number of cones by two, which is the number of ovules per cone.

### Ants as Pollinators: Pollen Load and Pollen Viability Tests

To quantify ant pollen load, we collected ten individual ants from each of eight common ant species observed consuming PDs in each of 15 female plants (*n* = 150 ants). The ants were collected from the cones using 50-mL Corning tubes, after recording whether they had been in contact with ovules’ micropylar tubes. To avoid cross-contamination of pollen load, a new clean tube was used for each insect. We induced cold anesthesia by placing the tube on ice and removed pollen non-destructively by dabbing the ants’ head, antennae, mesosoma, legs and gaster (the body parts that come into contact with the female cone) with a cube of fuchsin-stained gel (Kearns and Inouye, 1993). We then mounted and dissolved the pollen-containing gel on a microscope slide, to identify and quantify *E. triandra* pollen (with their characteristic Gnetales morphology, see above) under a microscope, by comparison to an *E. triandra* pollen control slide.

We conducted pollen germination assays to assess pollen viability after contact with the cuticle of three common ant visitors to female cones (56% of total visits, see Results) which showed the highest pollen load (*Camponotus mus*, *C. blandus*, and *Forelius chalybaeus*). Male cones were collected at anthesis and placed in vials together with live ants collected in the field; each vial containing one ant and 3–4 branches with 4–6 cones each for a total of ten individual workers of each ant species. Ten vials with cones and without ants were used as a control. Ants were kept in the vials for 24 h, allowing them to walk over pollen cones. Each ant was then held by the legs with insect forceps and rubbed on a drop of pollen germination solution (Brewbaker and Kwack, 1963) placed on a microscope slide to dislodge pollen from their bodies, and then released. A new slide was used for each ant. Control pollen that had not been in contact with ants was placed directly onto slides. Slides were kept covered inside Petri dishes at room temperature to avoid dehydration, and germination was monitored under a microscope every hour for 8 h, then every second hour until germination ceased. Pollen germinability was calculated as the percentage of pollen that germinated for each treatment (three ant species and control), by counting the number of pollen grains with and without pollen tubes.

### Ants as Plant Defenders: Quantification of Seed Cone Damage

To analyze whether ants protect seed cones against herbivory, we determined seed cone damage by counting the number of intact and damaged seed cones on the “Ant exclusion” and “Open pollination” (with ant access) treatments (*N* = 15 each). This was done while checking for seed viability on dissected seeds under a stereo microscope, by inspecting for signs of herbivory on the fleshy bracts and into the seeds. Seed cone damage included holes and missing parts in seeds and bracts, the presence of insect larvae or insect waste inside seeds, and the complete or partial destruction of embryos. The percentage of damaged seed cones was calculated as the ratio between the initial number of cones and the number of damaged cones × 100.

### Pollen Flow via Wind and Ant Visitation Rate as a Function of Distance

We used the exposed pollen traps on female plants described in section “Exclusion Experiments to Determine Pollination Mode” to estimate the amount of pollen reaching each experimental female plant as a function of their distance to the pollen source (nearest male).

To assess ant visitation rate to female cones and whether it varies as a function of the distance between female and male plants, we recorded ant visits on each of the 15 female plants used for the pollination mode experiments during November 2020. We used portable digital video cameras (Nikon Coolpix P900) rather than direct observations, which allowed for a less intrusive method of recording ant behavior (Gilpin et al., 2017). Cameras were positioned on tripods by one branch at each experimental plant, where 10 cones had been marked with a strip of tape, and set up to film for 30-min. We conducted five 30-min censuses on each plant (150-min per plant, and a total of 75 censuses on 15 plants), distributed during the morning (from 0700 to 1,000 h) and the afternoon (from 1,700 to 1,900 h) under suitable weather conditions (sunny, with low wind speed). We used the video recordings to count the number of times an ant visited individual cones during each 30 min period. Only ants consuming PDs or contacting the micropylar tubes of the ovules were considered cone visitors, without discriminating between same or different individual ant visitor. Ant visitation rate was calculated for each census as the number of visits per cone in a 30 min interval, averaged for each plant.

### Dynamics of Pollination Drop Secretion

To study the dynamics of PD secretion, ten branches (each bearing circa 30 cones) from five female plants were collected in the field at the beginning of the pollination period (October 2020) and kept in jars with water in the laboratory (25°C and 50–70% relative humidity). Preliminary observations showed that ovules secrete pollination drops overnight under those conditions. This procedure was preferred over direct field observations to avoid the risk of having rain wash the PDs, and for ease of observation under a dissecting microscope over extended periods. Cones that had produced PDs overnight were marked with tape (*n* = 60), PDs were gently removed from half of the cones using filter paper, and presence of newly secreted PDs was recorded every 6 h. PD volume was determined at each time interval as (4/3) πr^3^ (where r is the radius of a sphere). When a PD reached its maximum volume (no changes observed within a 6 h interval), it was removed using the same procedure and newly observed every 6 h. This removal procedure was repeated until no new PDs were secreted. Fresh pollen was collected from male cones with an entomological pin (> 100 pollen grains) and applied onto the PDs of the other half of the female cones by gently touching them with the pin. PDs were observed every 6 h the first day, and subsequently every 24 h until no further change was observed.

### Statistical Analyses

Ant pollen load (the number of pollen grains found on an individual) of different ant species were analyzed by fitting Generalized Linear Models (GLMs) with Poisson error distribution, as the data did not satisfy normality nor homogeneity assumptions. When differences among species were significant, we used *post hoc* Tukey’s pairwise comparisons. Differences in the percentage of germinated pollen attached to ants’ bodies among treatments (three ant species and control) were analyzed by means of GLMs with binomial error distribution followed by *post hoc* Tukey’s test, as the response variable was in percentage. To test whether the proportion of seed set was affected by the pollination-exclusion treatments, we fitted Generalized Linear Mixed Models (GLMMs) with binomial error distribution, as the response variable was in percentages. The fixed variables were treatments (Open, Wind only, and Ant exclusion), distance to the nearest male plant was included as a continuous predictor, and plant IDs as a random effect. When differences among treatments were significant, we used *post hoc* Tukey’s test. When the interaction term treatment ^∗^ distance was significant, the effect of distance on seed set was further analyzed separately for each treatment. As the effect of distance on seed set fitted an exponential decay relationship, we log_10_ (x + 1) transformed seed set and distance variables prior to performing the analyses. By linearizing the data, we were able to use simple linear regression to test the relationship between distance and seed set. Differences in seed cone damage between Open access and Ant exclusion treatments were analyzed using GLMM with binomial distribution, including distance as a continuous predictor and plant ID as random effect. The number of pollen grains per slide was compared between paired bagged and open pollen traps using a non-parametric Wilcoxon matched pair test, as the data did not meet assumptions of normality. The effects of distance on pollen transport by wind and ant visitation rate were analyzed using linear regressions on log_10_ (x + 1) transformed data.

Statistical analyses were performed in R version 3.5.2 (R Core Team, 2020). We used the *lmerTest* package for GLMs analyses (Kuznetsova et al., 2017) and the function *lsmeans* in the *emmeans* package for *post hoc* Tukey’s pairwise comparisons (Length, 2018). A multi-model selection based on Akaike’s information criterion corrected for small samples (AICc) was used to search for the most parsimonious models (the best model was the one with the lowest AIC value, models with ΔAICc ≤ 2 were considered equivalent). Model selection was made using the “*dredge*” function in the *MuMIn* package (Bartoń, 2019). Statistical analyses were considered significant at a *P*-value < 0.05.

## Results

### Ants Are the Main Visitor of *Ephedra triandra* Female Cones

Our observations and exclusion treatments demonstrated that male cones of *E. triandra* lack abortive ovules and hence do not produce pollination drops. No potential insect pollinators (ants included), were observed in direct contact with male cones. PDs were observed only on *E. triandra* female cones where insects had been excluded.

Ants (Hymenoptera: Formicidae) were the main female cone visitor, accounting for 99.89% of total visits (3966 visits on the 15 plants). At least eight ant species belonging to four subfamilies were observed consuming PDs (Figures 1C–F and Supplementary Table 1). The most abundant species was *Camponotus mus* (relative abundance 28.24%), followed by *Forelius chalybaeus* (21.81%) and *Brachymyrmex patagonicus* (18.71%). The solitary foragers *Pseudomyrmex maculatus* and *Cephalotes bruchi* were the least abundant; a single individual was typically found per plant and census. All other species consisted of more than five individuals per plant during each census, and displayed collective foraging behavior (recruiting nestmates when encountering a valuable food resource). The most frequent visitor was *B. patagonicus* (found on 80% of the plants), followed by *F. chalybaeus* and *P. maculatus* (60%).

Field observations showed that ants searched for PDs by systematically walking from cone to cone within a plant (see Supplementary Video 1). Ants inserted their mouth apparatus into the micropylar tube even when no PDs were visible to the naked eye. Patrolling ants were observed consistently across the whole pollination period, until cones began to mature. The co-occurrence of ant species on the same plant was common, and appeared to result from dominance hierarchies of the ant assemblage via competitive exclusion. Recruitment ants (those that enlist nestmates to new food sources), like *Camponotus* spp. and *Forelius* spp., were never found foraging together on the same plant. These ants did co-occur, however, with less aggressive solitary forager species like *Cephalotes* sp. and *Pseudomyrmex* sp. Visitation to female cones by non-ant insects was rare (Supplementary Table 1); and since they were not seen in direct contact with the cones, they were not considered potential pollinators.

### *E. triandra* Pollination Mode Is Ambophilous

**FIGURE 2:**
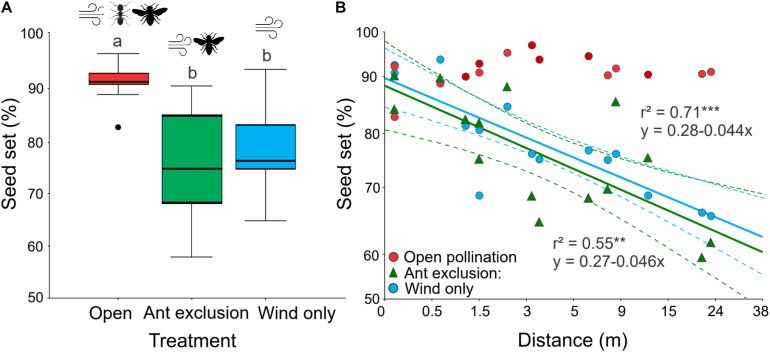
The effect of pollination treatment and distance to the nearest male on seed set in *Ephedra triandra*. **(A)** Percentage of mature seeds relative to the number of initial ovules under three pollination treatments (*N* = 15 plants). Boxes show median (line), 25th and 75th percentiles, whiskers encompass the range of values, and dots represent outliers. Different letters indicate significant differences (*post hoc* Tukey’s test, *P* < 0.001). **(B)** The effect of distance to the nearest male on seed set for the different pollination treatments (*N* = 15 for each treatment). Lines show significant linear regressions with 95% confidence intervals (dashed lines) on log_10_ (x + 1) transformed values. Significance: ***P < 0.0005, **P < 0.005. Note the log_10_ scale used in both axes. Values are shown back-transformed for clarity.

Seed set was significantly affected by pollination treatment (GLM: χ2 = 11.448, *df* = 2, *P* < 0.001; Table 1 and Supplementary Tables 2, 3). Ants improved seed production in *E. triandra* by 13% overall, with wind pollination being the main contributor (80% of seed set in Wind only treatment). There was no significant difference in the number of pollen grains found in open versus bagged pollen traps (*Z* = 1.376, *P* = 0.168, *N* = 60), suggesting that mesh bags did not impose a physical barrier to the transport of wind-born pollen. The Open pollination treatment (with free ant access) resulted in significantly more seeds than both ant exclusion treatments (Wind only and Ant exclusion) (*post hoc* Tukey’s test, *P* < 0.001; Figure 2A). Wind only and Ant exclusion treatments (with access to winged insects) were not significantly different (*post hoc* Tukey’s test, *P* = 0.47), suggesting that winged insects did not contribute significantly to pollination. Both the distance to the nearest male plant and the distance by treatment terms were statistically significant (GLM: distance: χ^2^ = 10.844, *df* = 1, *P* < 0.001; distance ^∗^ treatment: χ^2^ = 11.968, *df* = 5, *P* < 0.001), indicating that the effect of distance on seed set differed among treatments. In treatments where ants were excluded that depended exclusively on airborne pollen for pollination (Wind only and Ant exclusion groups), seed set decreased linearly with increasing distance from the nearest pollen donor [Wind only: *F*_(1, 13)_ = 32.40, *r*^2^ = 0.71, *P* < 0.0001; Ant exclusion: *F*_(1, 13)_ = 15.91, *r*^2^ = 0.55, *P* < 0.005; Figure 2B], with no significant differences in their regression slopes (*P* < 0.005). In Open pollination treatments, with access to ants and airborne pollen, seed set was not significantly affected by distance to nearest male (F_1, 13_ = 0.997, *P* = 0.336). Total exclusion treatments did not produce any seed, suggesting the absence of apomixis in this system.

**TABLE 1:** Statistical parameters of the best-fit Generalized Linear Models (GLMs) and Generalized Linear Mixed Models (GLMMs, including random effects), selected based on small sample corrected Akaike’s Information Criterion (AICc) for the different response variables.

**Response variable**	**Predictor variable**	**Random**	**Error distribution**	**Link**	**χ ^2^**	**df**	***P***	**AICc**
Seed set (%)	Treatment	Plant ID	Binomial	Logit	11.448	2	< 0.001*	634.8
	Distance				10.844	1	< 0.001*	
	Distance * Treatment				11.968	5	< 0.001*	
Pollen germination (%)	Treatment	_	Binomial	Logit	59.318	3	< 0.0001*	162.5
Pollen load	Ant species	Plant ID	Poisson	Log	283.36	3	< 0.0001*	1139.8
Seed cone damage (%)	Treatment	Plant ID	Binomial	Logit	0.008	1	0.926	2.29
	Distance				0	1	0.995	
	Distance * Treatment				5.753	2	0.056	

df = degrees of freedom. *Significant P-values.

### Ants Carry Pollen Without Affecting Its Viability

**FIGURE 3:**
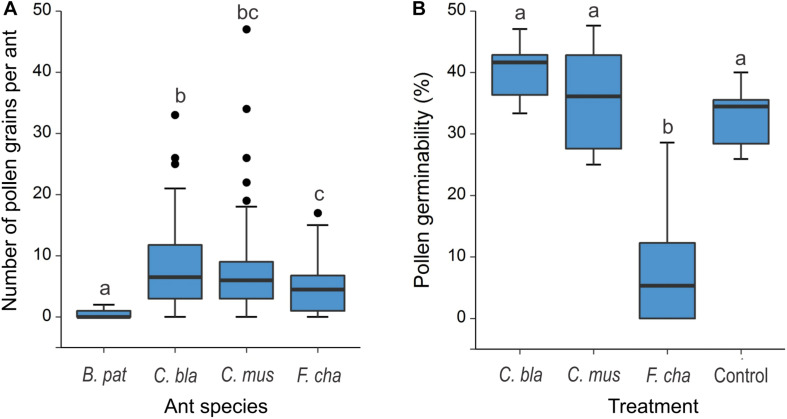
Pollen load and pollen grain germinability after contact with ants foraging on the pollination drops of *Ephedra triandra* female plants. **(A)** Number of pollen grains per individual on four ant species (*Brachymyrmex patagonicus n* = 24, *Camponotus blandus n* = 30, *C. mus n* = 73 and *Forelius chalybaeus n* = 14 individuals). **(B)** Pollen germinability (percentage of pollen grains that grow a pollen tube) for three ant species carrying the highest pollen load, compared to Control pollen, collected directly from male cones without contacting ants (*N* = 10 per treatment). Boxes show median (line), 25th and 75th percentiles. Whiskers encompass the largest and smallest values, and dots show outliers. Different letters indicate significant differences in *post hoc* Tukey’s test.

*E. triandra* pollen was found on the head, mesosoma and legs of *C. blandus, C. punctulatus, C. mus, B. patagonicus*, and *F. chalybaeus* collected on female cones (Figures 1E,F). No pollen grains were found on *Ce. bruchi* and *P. maculatus*. GLM analysis were conducted on the main visitors *C. blandus*, *C. mus*, *B. patagonicus* and *F. chalybeus*, not on species with lower sample size. Pollen load differed significantly between ant species (GLM: χ^2^ = 283.36, *df* = 3, *P* < 0.0001; Table 1 and Supplementary Table 2). Large *Camponotus* specimens (*C. mus*: 7.52 ± 7.55, *n* = 73 and *C. blandus*: 9.03 ± 8.49, *n* = 30; means ± SD) and the massive recruiter *F. chalybeus* (5.28 ± 5.38, *n* = 14) carried more pollen grains than the smaller *B. patagonicus* (0.37 ± 0.57, *n* = 24; *post hoc* Tukey’s test *P* < 0.0001, Figure 3A and Supplementary Table 4). Most of the pollen load was readily distinguished as belonging to *E. triandra* (>90%), angiosperm pollen was also present in lesser amounts. Pollen germination differed significantly between treatments (GLM: χ^2^ = 59.138, *df* = 3, *P* < 0.0001, Figure 3B, Table 1, and Supplementary Tables 2, 5). Pollen carried by *C. blandus* and *C. mus* showed no differences in their germination success (41.45 ± 6.68 and 35.55 ± 8.82% of, respectively, means ± SD, *N* = 10, *P* = 0.58) compared to the control treatment (37.14 ± 9.87%, *N* = 10, *P* = 0.56 and *P* = 0.99, respectively). In contrast, contact with *F. chalybaeus* ants reduced pollen germination by four to five times, compared to *Camponotus* spp. and control treatments (8.44 ± 10.13%, *N* = 10, *P* < 0.001).

### Ants Have no Effect on Seed Cone Damage

Evidence of damage to seed cones (fleshy bracts) was low overall (324 out of 2,142 cones, or 15%) in *E. triandra* female plants and it was not affected by the presence of ants. We found no significant effect of treatment (Open or Ant exclusion) in the percentage of damaged cones (GLM: χ2 = 0.008, *df* = 1, *P* = 0.926; Table 1 and Supplementary Table 2).

### Decreased Pollen Flow and Increased Ant Visitation Farther From Pollen Sources

**FIGURE 4:**
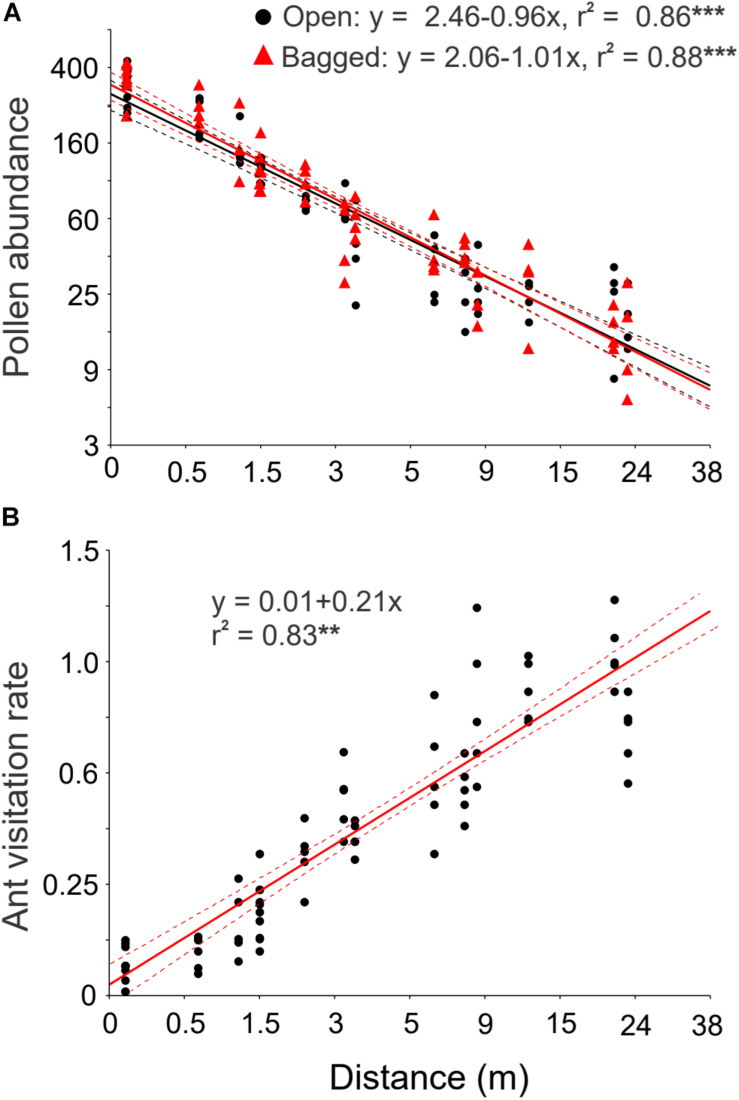
The effect of distance to the nearest male on pollen flow by wind and on the rate of ant visitation to *Ephedra triandra* female plants. **(A)** Pollen abundance (number of pollen grains per slide) in pollen traps placed on female plants at increasing distance to the nearest male plant. Open pollen traps (black circles) and traps enclosed in mesh bags (red triangles), to control for mesh effects (*n* = 60 pollen traps per treatment). **(B)** Ant visitation rate to female cones (number of ant visits per cone in 30 min) at increasing distance from a male plant (*n* = 75). Lines show significant linear regressions with 95% confidence intervals (dashed lines) on log_10_ (x + 1) transformed values. Significance: **P < 0.001, ***P < 0.0001. Note the log_10_ scale used in both axes. Values are shown back-transformed for clarity.

Pollen abundance on pollen traps decreased linearly with increasing distance between the focal female and its nearest male plant [linear regression: Open: *F*_(1, 58)_ = 120.61, *r*^2^ = 0.86, *P* < 0.0001; Bagged: *F*_(1, 58)_ = 126.99, *r*^2^ = 0.88, *P* < 0.0001, Figure 4A]. The number of pollen grains found on pollen traps was on average 20 times lower at the maximal distance between a female plant and the nearest male plant (23 m) compared to the minimal distance for both treatments.

The frequency of ant visits to female cones increased linearly with increasing distance to the nearest male plant [*F*_(1, 73)_ = 1.324, *r*^2^ = 0.83, *P* < 0.001, Figure 4B]. Ants on isolated female plants showed as much as 24-fold more visits compared to those closest to a male plant (from 0.043 ± 0.03 at 0.1 m to 1.04 ± 0.14 visits cone^–1^ 30 min^–1^ at 23 m, means ± SD, *n* = 75).

### Pollination Drops Are Secreted Repeatedly Until Pollination

**FIGURE 5:**
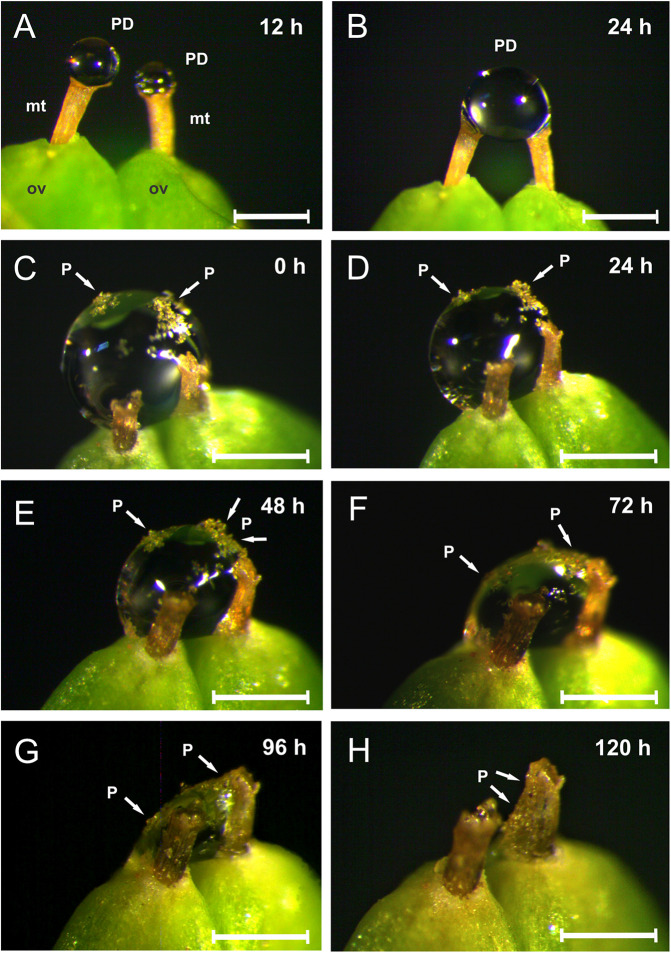
The dynamics of pollination drop secretion in *Ephedra triandra* (under laboratory conditions). **(A)** Two pollination drops (PD) newly formed on micropylar tubes (mt) of the two ovules (ov) 12 h after manual removal, and **(B)** fused into one droplet after 24 h. **(C–H)** PD withdrawal in artificially pollinated cones. **(H)** PD completely withdrawn after 120 h:, residual PDs and pollen grains can be seen on the rim of the micropylar tube opening, and along its exterior. P = pollen grains. Scale bars: 0.5 mm.

Under laboratory conditions, PDs were newly secreted between five and 10 times over consecutive days following manual removal, gradually accumulating on the micropyle until they reached their maximum volume, within 24 h. The two droplets (from two adjacent ovules) often fused into one (maximum volume = 0.56 ± 0.035 μl, mean ± SD, *N* = 17), otherwise, each single drop had approximately half that volume (0.37 ± 0.06 μl, mean ± SD, *N* = 13) (Figures 5A,B). Manually pollinated PDs showed decreased volume by 24 h, and then gradually withdrew until they completely disappeared after 4–5 days, when no further PD secretion was observed. Following complete withdrawal, residual PD with pollen grains was observed on the rim and along the exterior of the micropylar tube (Figures 5C–H).

## Discussion

Our study provides experimental evidence for an ambophilous pollination system (by wind and insects) in the dioecious gymnosperm *Ephedra triandra.* While wind remained the main pollen vector overall (13% vs. 80% of seed set), ants significantly contributed to plant reproductive success in pollen-limited females. Even though the contribution of ants to pollination in female plants closest to a male plant was not significant, ants enhanced seed set by 30% at farther distances from a pollen source, compared to plants pollinated only by wind (90% vs. 60% of seed set, respectively). Moreover, our observations suggest that ants enhance pollination success while consuming PDs through frequent contact with the micropylar tubes of ovules. Hence, we propose that ants enhance reproductive assurance in pollen-limited plants by compensating for the negative effect of increased distance between the sexes on pollen flow.

Our results do not support the existence of a protective or double mutualism (combining pollination and plant defense) between *E. triandra* and its ant visitors. We found no difference in seed cone herbivore damage between plants excluded from ants and those accessible. Ant defensive mutualisms mediated by sugary secretions are especially variable, their net outcome typically being context-dependent (Chamberlain and Holland, 2009; Rosumek et al., 2009). For instance, it is possible that when the rate of herbivory is low (as in our case) the benefit of ant defense could only be detected over extended time periods (Heil et al., 2001). Longer-term studies are therefore needed to further investigate a potential ant-plant protective mutualism in *E. triandra*.

### The Distance Effect

We found that increased distance between female and male plants negatively affected the transport of pollen by wind, while indirectly enhancing ant pollination by increasing visitation to isolated females with continuous PD secretion. Both seed set in ant exclusion treatments and the amount of pollen trapped on experimental slides decreased with increasing distance to a pollen donor. Where female and male plant individuals intertwine (distances ≤ 0.1 m), pollen rain may lead to pollination simply by gravitational pollen transfer (Di-Giovanni and Kevan, 1991). A decrease in pollen dispersal with increasing distance from a pollen donor has been documented in other wind-pollinated species (e.g., Robledo-Arnuncio and Gil, 2005; Bai et al., 2007), including *Ephedra* (Meeuse et al., 1990; Bolinder et al., 2016). Here, we add to this pattern the observation that distance to a pollen donor can affect ant visitation rates, with measurable implications to increased plant reproductive success.

Evidence presented here on enhanced ant interaction as a function of distance to the nearest male likely relates to the dynamics of PD secretion, combined with ant behavior. Up to three cycles of PD secretion have been documented in *E. distachya* after experimental removal (Moussel, 1980), while we observed up to ten. Assuming that ants completely remove PDs, and that female plants at increasing distance from males receive less airborne pollen, we can expect that those plants will secrete more PDs (over an extended period of time). Ants are social insects that live in colonies and recruit nestmates to valuable food sources (Hölldobler and Wilson, 1990). In protective mutualisms mediated by sugary secretions like extrafloral nectar, ant visitation increases with increased nectar production (Lange et al., 2017). These types of behavior could explain why ant visitation is higher as distance to pollen source increases, since colony response might be stimulated by the repeated secretion of unpollinated PDs.

After PD withdrawal, we observed pollen remnants on the surface of micropylar tubes, which ants are likely to transport from cone to cone while searching for PDs, thus acting as pollinators. A comparable secondary pollination mechanism has been described for ants transferring *Scleranthus perennis* pollen (Caryophyllaceae) previously deposited by a primary pollinator to new flowers in a carryover sequence (Svensson, 1986). Additionally, pollen could be deposited on the micropylar tubes by ants while sucking the drops (Carafa et al., 1992). Alternatively, repeated secretion by ovules following PD consumption might scavenge pollen left by ants on the micropylar tube rim (von Aderkas et al., 2018).

### Ant Species-Specific Traits Promote Pollination

Ants have been reported as frequent insect visitors and potential pollinators in other *Ephedra*: in the exclusively insect-pollinated *E. foeminea* (Bolinder et al., 2016), in ambophilous *E. distachya* (Moussel, 1980; Bolinder et al., 2016) and in *E. aphylla* (Meeuse et al., 1990), as well as on presumed anemophilous *E. helvetica* (Ziegler, 1959). Evidence presented here suggests that not all ant visitors may be considered equal when evaluating potential pollinators. Genus- or species- level ant traits that consistently favor high visitation rates (such as collective foraging), suitable pollen load (such as large body size) and lack of negative effects on pollen viability (i.e., lack of metapleural glands) will be important considerations.

The main reason ants are arguably poor pollinators is that they produce antimicrobial secretions from their metapleural glands (MG). Although the main function of MGs is antiseptic, preventing fungal growth inside the nests (Hölldobler and Wilson, 1990), they can also have detrimental side effects on pollen viability (Beattie et al., 1984). Metapleural glands are an ant innovation that has been lost repeatedly and is absent in *Camponotus* (Yek and Mueller, 2011), the most abundant visitors of *E. triandra*. Indeed, *Camponotus* ants in our study did not reduce pollen germinability, in agreement with results reported for other *Camponotus* species (Delnevo et al., 2020, but see de Vega et al., 2009, for a counterexample) including in *E. foeminea*, where an actual increase in pollen germinability was observed after contact with these ants (Bolinder et al., 2016). Moreover, species of *Camponotus* have been frequently associated with pollination in dry habitats (de Vega et al., 2014; Del-Claro et al., 2019). *Camponotus mus* and *C. blandus* worker ants in our study, also known as sugar or carpenter ants, are relatively large (7.5–13 mm long, Aranda-Rickert and Fracchia, 2011b), potentially leading to greater pollen collection because of a larger surface area (Müller et al., 2006), and hairy, which might favor pollen attachment (Goulnik et al., 2020). These ants forage for nectar and honeydew and prey or scavenge on arthropods (Fernández, 2003), monopolizing resources (Aranda-Rickert and Fracchia, 2011b). In contrast to solitary foragers such as *Pseudomyrmex* spp., the collective foraging behavior of *Camponotus* ants potentially further increases their pollination efficiency, since they actively recruit nestmates when encountering a stationary and renewable carbohydrate resource (Carroll and Janzen, 1973). Together with their short-term foraging specialization and concentrated activity on a single plant (“floral fidelity,” Brosi, 2016), these morphological and behavioral traits suggest that *Camponotus* spp. could contribute disproportionally to *E. triandra* pollination compared to the other ant visitors.

**FIGURE 6:**
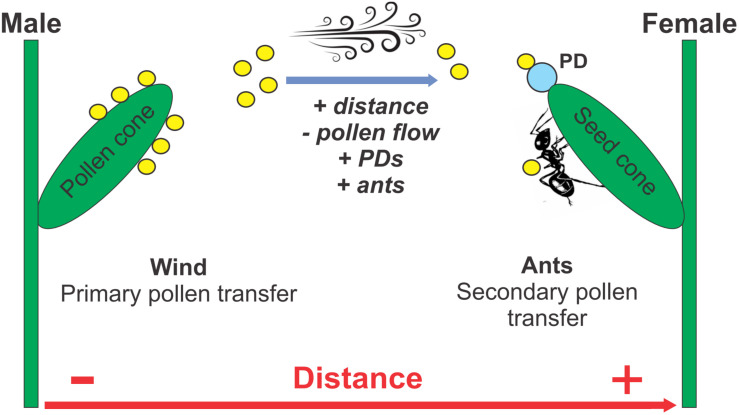
Proposed model for the combined wind and ant pollination of dioecious *Ephedra triandra*. Wind is the primary pollen vector, carrying pollen grains (yellow circles) from male to female plants. When pollen lands on pollination drops (PD) secreted by ovules, fertilization is achieved. Ants become secondary pollen vectors when they redirect pollen on their bodies to the ovules while feeding on PDs. As the distance between female plants and pollen donors increases (+), airborne pollen flow decreases (–), more PDs are secreted (+), and more ants are recruited to collect the PDs (+).

Male cone morphology has been used as indirect evidence to infer pollination biology in Gnetales. Animal-pollinated species typically have abortive ovules that produce PDs and attract pollinators in male cones, while wind-pollinated species lack them (Bolinder et al., 2016). However, a mechanism of insect pollination has not been suggested for ambophilous *Ephedra* that lack PDs on male cones (Bino et al., 1984a; Meeuse et al., 1990). Here, we propose a mechanism for secondary pollination by ants in *E. triandra* (Figure 6) as a working hypothesis for ambophily in other Gnetales potentially applicable to other dioecious plants lacking pollinator reward in males. In systems where wind is the primary pollen vector, ants may act as secondary pollinators by subsequently delivering pollen grains that previously landed on female plants or on themselves. Because ants actively and persistently patrol female plants in search of PDs as reward, frequent contact with the micropylar end of ovules leads to a more precise placement of pollen grains, closer to the site of fertilization.

## Conclusion

Our study constitutes the first experimental quantification of distance-dependent contribution of ants to pollination in a dioecious, primarily wind-pollinated gymnosperm. Field observations combined with experiments support an ambophilous pollination mode for *Ephedra triandra* where wind plays a larger role in plant fertilization success than ants, yet the relative contribution of ants as pollinators increases significantly (up to 30%) as females are farther away from a pollen source. The interaction between ants and *E. triandra* is mediated by pollination drops as a reward, with no detectable short-term effect on plant protection against herbivores. *Camponotus* spp. in our study have behavioral and physical traits that favor pollination, suggesting that certain ant lineages likely contribute disproportionally to this type of previously neglected interaction.

## Data Availability Statement

The original contributions presented in the study are included in the article/Supplementary Material, further inquiries can be directed to the corresponding author/s.

## Author Contributions

AA-R and VD conceived and designed the research. VD obtained the funding. AA-R and NY led data-analysis. MB coordinated logistics. JT provided entomology and microscopy resources. AA-R and VD led the writing, with contributions from the other authors. All authors conducted fieldwork.

## Conflict of Interest

The authors declare that the research was conducted in the absence of any commercial or financial relationships that could be construed as a potential conflict of interest.

## Publisher’s Note

All claims expressed in this article are solely those of the authors and do not necessarily represent those of their affiliated organizations, or those of the publisher, the editors and the reviewers. Any product that may be evaluated in this article, or claim that may be made by its manufacturer, is not guaranteed or endorsed by the publisher.
