# Supplementary material for: Distance Dependent Contribution of Ants to Pollination but Not Defense in a Dioecious, Ambophilous Gymnosperm
Source: Front Plant Sci. 2021 Sep 8;12:722405. doi: 10.3389/fpls.2021.722405 (PMC8459830; doi:10.3389/fpls.2021.722405)
Supplement: Supplementary file 1 [file Table_1.docx]

Supplementary Material

**Supplementary Table 1.** Absolute and relative abundance of insect visitors on female cones of *Ephedra triandra*. Frequency refers to the number of plants in which each insect species was observed (N = 15 plants).

| **Visitor** | **Absolute abundance** | **Relative abundance (%)** | **Frequency** |
| --- | --- | --- | --- |
| **Order Hymenoptera** |  |  |  |
| **Family Formicidae** |  |  |  |
| Subfamily Dolichoderinae |  |  |  |
| *Forelius albiventris* Forel | 524 | 13.21 | 6 |
| *Forelius chalybaeus* Emery | 865 | 21.81 | 9 |
| Subfamily Formicinae |  |  |  |
| *Camponotus blandus* Smith F. | 253 | 6.37 | 3 |
| *Camponotus mus* Roger | 1120 | 28.24 | 6 |
| *Camponotus punctulatus* Mayr | 76 | 1.91 | 3 |
| *Brachymyrmex patagonicus* Mayr | 742 | 18.71 | 12 |
| Subfamily Myrmicinae |  |  |  |
| *Cephalotes bruchi* Forel | 15 | 0.37 | 6 |
| *Pheidole bergi* Mayr | 340 | 8.57 | 6 |
| Subfamily Pseudomyrmicinae |  |  |  |
| *Pseudomyrmex maculatus* Smith | 28 | 0.7 | 9 |
| **Family Mutillidae** |  |  |  |
| *Tallium* sp. | 1 | 0.02 | 1 |
| **Order Coleoptera** |  |  |  |
| **Family Anthicidae** |  |  |  |
| Anthicidae spp. | 2 | 0.05 | 1 |
| Total | 3966 | 100 | 15 |
