# Supplementary material for: Distance Dependent Contribution of Ants to Pollination but Not Defense in a Dioecious, Ambophilous Gymnosperm
Source: Front Plant Sci. 2021 Sep 8;12:722405. doi: 10.3389/fpls.2021.722405 (PMC8459830; doi:10.3389/fpls.2021.722405)
Supplement: Supplementary file 2 [file Table_2.DOCX]

Supplementary Material

**Supplementary Table 2.** Selection of GLM (Generalized Linear Models) and GLMMs (Generalized Linear Mixed Models) for the different response variables. Models are ranked by AICc (Akaike’s information criterion corrected for small samples).

| **Response variable** | **Model** | **df** | **AICc** | **logLik** | **ΔAICc** |
| --- | --- | --- | --- | --- | --- |
| Pollen load (GLM) | **1:** Number pollen grains ~ species | 4 | 1139.8 | -565.737 | 0.00 |
|  | **2:** Number pollen grains ~ 1 | 1 | 1416.9 | -707.416 | 277.09 |
| Pollen germination (%) (GLM) | **1:** pollen germination ~ treatment | 4 | 162.5 | -76.665 | 0.00 |
|  | **2:** pollen germination ~ 1 | 1 | 214.8 | -106.324 | 52.28 |
| Seed set (%) (GLMM, random plant ID) | **1:** seed set ~ treatment + distance + distance * treatment | 5 | 634.8 | -311.623 | 0.00 |
|  | **2**: seed set ~ treatment + distance | 4 | 644.1 | -317.550 | 9.32 |
|  | **3:** seed set ~ treatment | 3 | 657.3 | -325.360 | 22.53 |
|  | **4:** seed set ~ distance | 3 | 684.6 | -338.989 | 49.78 |
|  | **5:** seed set ~ 1 | 2 | 697.5 | -346.619 | 62.74 |
| Seed cone damage (%) (GLMM, random plant ID) | **1:** seed damage ~ 1 | 2 | 219.4 | -107.454 | 0.00 |
|  | **2:** seed damage ~ treatment + distance + distance * treatment | 5 | 221.6 | -104.573 | 2.29 |
|  | **3**: seed damage ~ treatment | 3 | 221.8 | -325.360 | 2.47 |
