# Supplementary material for: Distance Dependent Contribution of Ants to Pollination but Not Defense in a Dioecious, Ambophilous Gymnosperm
Source: Front Plant Sci. 2021 Sep 8;12:722405. doi: 10.3389/fpls.2021.722405 (PMC8459830; doi:10.3389/fpls.2021.722405)
Supplement: Supplementary file 3 [file Table_3.DOCX]

Supplementary Material

**Supplementary Table 3.** Pollen load on ants. Post-hoc pairwise comparison (Tukey method)

between pollen load on four ant species. * Significant P-values.

| **Contrast** | **Estimate** | **SE** | **Z-ratio** | **P-value** |
| --- | --- | --- | --- | --- |
| *B. patagonicus* - *C. blandus* | -3.182 | 0.3388 | -9.391 | < 0.0001* |
| *B. patagonicus* - *C. mus* | -2.998 | 0.3360 | -8.923 | < 0.0001* |
| B*. patagonicus - F. chalybaeus* | -2.646 | 0.3530 | -7.495 | < 0.0001* |
| *C. blandus - C. mus* | 0.183 | 0.0742 | 2.469 | 0.0649 |
| *C. blandus - F. chalybaeus* | 0.536 | 0.1312 | 4.086 | 0.0003 |
| *C. mus - F. chalybaeus* | 0.353 | 0.1238 | 2.848 | 0.0229 |
