# Supplementary material for: Distance Dependent Contribution of Ants to Pollination but Not Defense in a Dioecious, Ambophilous Gymnosperm
Source: Front Plant Sci. 2021 Sep 8;12:722405. doi: 10.3389/fpls.2021.722405 (PMC8459830; doi:10.3389/fpls.2021.722405)
Supplement: Supplementary file 4 [file Table_4.DOCX]

Supplementary Material

**Supplementary Table 4.** Pollen germinability after contact with ants. Post-hoc pairwise comparison (Tukey method) between pollen load on three ant species and control. * Significant P-values.

| **Contrast** | **Estimate** | **SE** | **Z-ratio** | **P-value** |
| --- | --- | --- | --- | --- |
| *C. blandus - C. mus* | 0.2902 | 1.262 | 1.262 | 0.5875 |
| *C. blandus -* Control | 0.2466 | 0.190 | 1.300 | 0.5626 |
| *C. blandus – F. chalybaeus* | 2.2160 | 0.364 | 6.084 | < 0.0001* |
| *C. mus -* Control | -0.0436 | 0.200 | -0.218 | 0.9964 |
| *C. mus - F. chalybaeus* | 1.9258 | 0.370 | 5.208 | < 0.0001* |
| Control *- F. chalybaeus* | 1.9694 | 0.346 | 5.690 | < 0.0001* |
