# Supplementary material for: Distance Dependent Contribution of Ants to Pollination but Not Defense in a Dioecious, Ambophilous Gymnosperm
Source: Front Plant Sci. 2021 Sep 8;12:722405. doi: 10.3389/fpls.2021.722405 (PMC8459830; doi:10.3389/fpls.2021.722405)
Supplement: Supplementary file 5 [file Table_5.DOCX]

Supplementary Material

**Supplementary Table 5.** Seed set under different exclusion treatments. Post-hoc pairwise comparison (Tukey method) between AE (Ant Exclusion), W (Wind only) and C (Control, open pollination). * Significant P-values.

| **Contrast** | **Estimate** | **SE** | **Z-ratio** | **P-value** |
| --- | --- | --- | --- | --- |
| AE - C | -1.328 | 0.0944 | -14.066 | < 0.0001* |
| AE - W | -0.224 | 0.0738 | -3.031 | 0.0069 |
| C - W | 1.105 | 0.0963 | 11.472 | < 0.0001* |
